# OPERAS decision support system versus manual job coding: a quantitative analysis on coding time and inter-coder reliability

**DOI:** 10.1136/oemed-2024-109823

**Published:** 2025-06-13

**Authors:** Mathijs A Langezaal, Egon L van den Broek, Grégoire Rey, Nicole Le Moual, Corinne Pilorget, Marcel Goldberg, Roel Vermeulen, Susan Peters

**Affiliations:** 1 Population-Based Epidemiological Cohorts Unit UMS11, INSERM, Villejuif, France; 2 Department of Information and Computing Sciences, Utrecht University, Utrecht, The Netherlands; 3 France Cohortes UMS47, INSERM, Paris, France; 4 INSERM, Équipe d’Épidémiologie Respiratoire Intégrative, CESP, Université Paris-Saclay, UVSQ, Villejuif, France; 5 Santé publique France, Saint-Maurice, France; 6 Institute for Risk Assessment Sciences, Utrecht University, Utrecht, The Netherlands

**Keywords:** Artificial intelligence, Epidemiology, Occupational Health

## Abstract

**Objectives:**

The manual coding of job descriptions is time-consuming, expensive and requires expert knowledge. Decision support systems (DSS) provide a valuable alternative by offering automated suggestions that support decision-making, improving efficiency while allowing manual corrections to ensure reliability. However, this claim has not been proven with expert coders. This study aims to fill this omission by comparing manual with decision-supported coding, using the new DSS OPERAS.

**Methods:**

Five expert coders proficient in using the French classification systems for occupations PCS2003 and activity sectors NAF2008 each successively coded two subsets of job descriptions from the CONSTANCES cohort manually and using OPERAS. Subsequently, we assessed coding time and inter-coder reliability of assigning occupation and activity sector codes while accounting for individual differences and the perceived usability of OPERAS, measured using the System Usability Scale (SUS; range 0–100).

**Results:**

OPERAS usage substantially outperformed manual coding for all coders on both coding time and inter-coder reliability. The median job description coding time was 38 s using OPERAS versus 60.8 s while manually coding. Inter-coder reliability (in Cohen’s kappa) ranged 0.61–0.70 and 0.56–0.61 for the PCS, while ranging 0.38–0.61 and 0.34–0.61 for the NAF for OPERAS and manual coding, respectively. The average SUS score was 75.5, indicating good usability.

**Conclusions:**

Compared with manual coding, using OPERAS as DSS for occupational coding improved coding time and inter-coder reliability. Subsequent comparison studies could use OPERAS’ ISCO-88 and ISCO-68 classification models. Consequently, OPERAS facilitates large, harmonised job coding in large-scale occupational health research.

**WHAT IS ALREADY KNOWN ON THIS TOPIC**

- Manual occupational coding requires substantial time and expertise. Tools for automatic coding do exist; however, for accurate exposure assessment, humans are still required. Decision support systems (DSS) offer automated suggestions that support decision-making, improving efficiency while allowing manual corrections to ensure reliability. However, the claimed impact of DSS on the occupational coding process’s coding time and inter-coder reliability of assigning occupation and activity sector codes remains unproven with expert coders.

**WHAT THIS STUDY ADDS**

- We compare the OPERAS DSS to manual coding on both coding time and inter-coder reliability with five expert coders. Our results show that decision-supported coding with OPERAS significantly outperforms manual coding in both aspects.

**HOW THIS STUDY MIGHT AFFECT RESEARCH, PRACTICE OR POLICY**

- The DSS OPERAS significantly improves the coding time and reliability of job coding. As such, it potentially facilitates the uptake of large-scale occupational health research.

## Introduction

 Large-scale cohort and case–control studies play a crucial role in occupational epidemiology by assessing health effects associated with occupational exposures. One common method to assess these associations in such large-scale studies is reconstructing subjects’ job histories and, subsequently, linking them to job-exposure matrices.^1 2^ To reconstruct subjects’ job histories, respondents answer open-ended questionnaires containing questions about a respondent’s job, resulting in answers in a free-text format.^3^ Before effective use, these free-text job descriptions require standardisation, using occupational or industry classification systems like the International Standard Classification of Occupations.^4^ This process, usually performed manually, is time-consuming, expensive and requires expert knowledge.^5^ Moreover, expert coders typically have a limited monthly coding capacity of ~2700 codes.^6^

Tools have been developed to assist with the manual coding process^7 8^ and can even automate it entirely.^9^,^13^ However, accurate exposure assessment still requires human interaction,^14 15^ especially when the tools are applied to novel datasets.^16^ Additionally, humans can adapt their coding to specific industrial or geographical contexts. A valuable alternative to both manual and fully automated coding in occupational coding is artificial intelligence’s (AI’s) decision support systems (DSS) that provide automated suggestions to assist users in decision-making.^17^

DSS can generate recommendations for job descriptions, often accompanied by an estimated probability of correctness,^13 18 19^ potentially improving coding time. When suggestions are incorrect, expert coders can ensure reliability through manual corrections. Additionally, DSS promote consistency, as users tend to select the same codes for similar job descriptions, thereby enhancing both inter- and intra-coder reliability.^19^ In cases where the system’s confidence score is high and users trust its recommendations, suggestions can be processed automatically, with expert coders reviewing the remaining cases, accompanied by the provided suggestions. This approach is typically referred to as semi-automatic or computer-assisted coding.^20 21^ However, since manual review remains essential to ensure accurate exposure assessment,^6 14 20^ decision support plays a critical role in occupational coding by improving coding time through code suggestions while maintaining reliability through potential corrections.

While DSS hold the potential to improve the occupational coding processes’ coding time and inter-coder reliability, their impact on the occupational coding process has not been quantitatively assessed with expert coders.^13 21^ This study addresses this gap by evaluating OPERAS, a state-of-the-art DSS for epidemiological job coding.^13^ Using the same underlying data, this study compares OPERAS to manual coding, measuring differences in coding time and inter-coder reliability.

## Methods

To evaluate the effect of the usage of OPERAS on expert coders’ coding time and inter-coder reliability during the occupational coding process, we employed a within-subject design. Following this design, coders proficient in using the hierarchically structured PCS2003 and NAF2008 classifications coded two different, but equally sized and difficult-to-code subsets of job descriptions, both manually and using OPERAS successively. After coding, we compared coding time and inter-coder reliability. In the following paragraphs, the study design is explained in detail.

### Coders

Five French expert coders with occupational coding experience using the French PCS2003 and NAF2008 were recruited for this study. Their coding experience with the aforementioned classifications ranged from 6 months to 15 years. The last time they had coded using these classifications ranged from 0 to 12 months ago. None of the coders had prior experience with (semi-)automatic or decision-supported coding. All coders previously coded exclusively manually and used CAPS (http://www.CAPS-France.fr),^7^ a French web application designed to assist with finding PCS2003 and NAF2008 codes.

### Materials

Using questionnaires, we collected information on the coders’ PCS2003 and NAF2008 coding experience and experience with tools similar to OPERAS. We used the short-form Computer Proficiency Questionnaire (CPQ-12) to measure computer proficiency, as it could influence software usage performance.^22^ To assess OPERAS’ perceived usability, we used the French System Usability Scale (SUS).^23^ The SUS contains 10 questions regarding the perceived usability of a system, answered on a Likert scale from 1 (strongly disagree) to 5 (strongly agree). An adjective rating (eg, ‘poor’ or ‘excellent’) can be assigned based on the average SUS score of all participants.^24^

The job descriptions to be coded were selected from the CONSTANCES cohort, a French general-purpose cohort (adults aged 18–69) focused on occupational and environmental factors.^25^ The dataset included free-text descriptions of participants’ occupations and activity sectors, as well as the start and end year of a job, employment status (ie, salaried worker or self-employed), type of contract (ie, open-ended or fixed-term) and work-time schedule (ie, full-time or part-time). Expert coders manually coded this information into the French PCS2003 and NAF2008 classifications, which both use hierarchical coding structures to classify occupations and activity sectors, respectively. A PCS code consists of four levels of aggregation and contains three digits and a letter (eg, ‘211A’), whereas an NAF code has five levels of aggregation and contains four digits and a letter (eg, ‘1013A’). Here, each added character adds more detail about the described job or activity sector. The descriptive statistics of CONSTANCES’ job descriptions and their implications on the classification performance can be found in Langezaal *et al*.^13^

To ensure a fair comparison between OPERAS and manual coding, we developed two job description subsets of equal size and coding difficulty. Coding the same subset twice would be inadequate, as this would result in memorisation of the assigned code in the second condition (ie, coding method), negatively impacting validity. Additionally, an expert coder repeatedly coding the same job descriptions is not representative of real-world scenarios. Expert coders may have coding experience with descriptions from different populations, leading to differences in coding time due to their perceived coding difficulty of descriptions stemming from these worker groups. For example, one coder might be more familiar with coding descriptions from farmers (ie, codes from category ‘1’), while another more frequently codes descriptions from artisans (ie, codes from category ‘2’). Furthermore, at the least aggregated coding level, the detail and distinction between codes within the same higher-level group can vary. For instance, differentiating between code ‘472B’: ‘Surveyors, topographers’ and ‘472C’: ‘Quantity surveyors and various building and public worker technicians’ is more nuanced and could be perceived as more challenging than deciding between ‘431B’: ‘Psychiatric nurses’ and ‘431C’: ‘Nursery nurses’, where the distinction is clearer. Since perceived coding difficulty affects coding time, the overall perceived difficulty between subsets must be equal. Additionally, it is unknown which specific jobs will need to be classified in future, real-world OPERAS usage, where the system should be applicable across all job types. Hence, we included a wide range of descriptions in the subsets reflecting the overall French worker population. We achieved this via stratified random sampling from CONSTANCES, aligned with the 2017 French Census second-level PCS2003 distribution.^26^ We sampled 100 subjects, resulting in 326 job descriptions per subset.

To enable a statistical coding time comparison on an individual level, we measured coding time for each job description separately and subsequently compared coding time between conditions based on code pairs. A code pair consists of one description from one subset and one description from the other subset with the same perceived coding difficulty. These were developed by matching the PCS codes of job descriptions at the least aggregated coding level between the two subsets (eg, ‘421A’). If a match could not be found at the least aggregated level, pairs were formed using more aggregated code levels (eg, ‘421’, ‘42’, etc) until all job descriptions were matched. Subsequently, an expert coder (not involved in the present coding process) verified that both codes in each code pair are of equal coding difficulty to minimise differences in coding time between subsets attributable to variations in the job descriptions’ perceived coding difficulty.

OPERAS’ AI provides occupational and/or activity code classification suggestions for free-text job descriptions.^13^ OPERAS displays the information for each job description in a row format, with customisable and sortable columns to accommodate different coding strategies (see online supplemental figure S1). For each job description, OPERAS provides three code suggestions on the least aggregated coding level in a dropdown menu for each coding system. These suggestions were generated using the same data processing strategies and classification models as described in Langezaal *et al*.^13^ Hovering over a suggestion displays the available information from the coding index related to the suggested code. Each suggestion includes an estimated chance of correctness (ranging 0%–100%), allowing users to set a threshold for automatic coding.^13^ If suggestions are deemed incorrect by the user, OPERAS allows users to manually overwrite them. Within each row, OPERAS displays a checkbox clickable by users to indicate that the job description in that row is coded. When clicked, this checkbox saves a timestamp in OPERAS’ database for subsequent coding time analyses. For OPERAS coding, coders were provided with OPERAS containing one of the subsets. They were shown all occupational information present in the CONSTANCES dataset for those descriptions.

For manual coding, the coders were provided with an Excel file containing the job descriptions of one subset, showing all the same information present during OPERAS coding. Here, we developed a macro that saved a timestamp whenever the PCS or NAF field was edited for subsequent analyses. Similar to OPERAS, the columns were sortable to accommodate different coding workflows.

### Study design

This study employed a within-subject design, a method where the same participants are exposed to all experimental conditions, allowing direct comparisons within individuals.^27^ Here, five expert coders successively coded two subsets of job descriptions: one manually and one using OPERAS. This design was chosen to minimise potential biases in coding time measurements caused by differences between coders (eg, coding experience) between the two conditions. To account for fatigue and procedural effects, two coders started with manual coding, while three began with OPERAS coding. After the first session, the coders coded the other subset in the second session. Coders completed coding in their familiar work environment with breaks and in as many sittings as needed. This ensured a realistic occupational coding scenario and high ecological validity. The study procedure consists of four data collection stages:

1. Explanation of the study, informed consent and questionnaires assessing coding experience and computer proficiency.
2. Coding session 1.
3. Coding session 2.
4. Assessment of OPERAS’ perceived usability.

During the first stage, coders received information about the study’s objectives verbally and through an information letter. We instructed the coders to record their computer screens during both coding conditions to secure the accuracy of the data for subsequent analyses. After indicating their understanding of the procedure and contents of the study, they completed the informed consent form. Subsequently, questionnaires on the coders’ PCS2003 and NAF2008 coding experience, experience with tools similar to OPERAS, and the CPQ-12 were conducted. Using the aforementioned counterbalanced design, we assigned coders to one of the coding orders.

In stage 2, coders coded one of the two subsets, using either OPERAS or manual coding, based on their assigned coding order. In stage 3, they coded the other subset using the alternative method. Coders assigned to order 1 began with manual coding in the first session and used OPERAS in the second session, while those assigned to order 2 followed the opposite sequence. During the manual coding phase, coders received an Excel file containing one of the job description subsets. Coders coded each job description using PCS2003 and NAF2008. To replicate the expert coders’ normal coding workflow, they could modify the file for the manual coding process. During the OPERAS coding stage, coders coded the other subset of job descriptions into the PCS2003 and NAF2008 using OPERAS. Beforehand, coders received an explanation of OPERAS’ features and received instructions to code to the least aggregated coding level, unless the data only supported coding to a lower level due to missing or incomplete information. After both coding stages had concluded, the SUS was conducted to assess OPERAS’ perceived usability.

### Data collection and statistical analysis

Since all coders performed the coding procedures in their natural work environment, all data were collected remotely. We measured coding time in both conditions using the generated timestamps in OPERAS and Excel. The time to code each description is calculated as the difference between consecutive timestamps in full seconds. For example, if code A is completed at 12:15:41 and code B at 12:17:43, the time to code code B is 122 s. Consequently, if codes are copied and therefore coded within the same second, this is reflected as a coding time of 0 s.

All recordings were manually reviewed to correct timestamps if needed. For both coding conditions, this included checking whether a coder continued working on a description/code after a timestamp was created. In such cases, timestamps were corrected to one-tenth of a second.

To allow for an overall comparison between OPERAS and manual coding time, we averaged the coding efficiencies of individual job descriptions of each participant. The Shapiro-Wilk test for normality reveals that the data for both OPERAS (W(5)=0.956, p=0.753) and manual coding (W(5)=0.981, p=0.942) are normally distributed. Consequently, we used a paired samples t-test to compare coding time between OPERAS and manual coding. For all coders separately, the distribution plots of the coding time appeared neither normal nor symmetrical. Consequently, we applied the non-parametric paired-samples sign test to compare coding time between OPERAS and manual coding on an individual level.^28^ To assess whether the use of DSS significantly influences code selection compared with manual coding, we conducted a χ^2^ test to compare the distribution of second-level PCS and NAF codes between OPERAS and manual coding. We also conducted ad-hoc analyses to explore potential interactions between overall coding time and several covariates: coding experience, time since last coding using PCS2003 and NAF2008, computer literacy (measured by CPQ-12) and perceived usability (assessed using the SUS).

We calculated inter-coder reliability using Cohen’s kappa, evaluating each condition in which coders used the same subset.^29^ Additionally, we assessed agreement between each coder and the ‘gold standard’ manually coded job descriptions from CONSTANCES. For all evaluations, we compared the inter-coder reliability between OPERAS and manual coding for both the PCS and NAF separately on each coding level.

## Results

We obtained 1630 records of coded job descriptions. All coders provided their final code for each job description, resulting in an inter-coder reliability comparison on all records. After manually checking screen recordings of the coding, 1204 out of 1630 (73.8%) valid records remained for the coding time comparison due to missing screen recordings.

**Figure 1:**
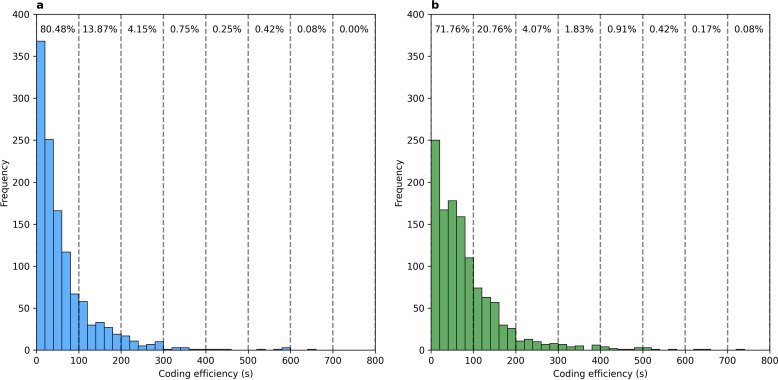
Distribution of the coding time for individual job descriptions of expert coders using (a) OPERAS and (b) manual coding in seconds. Vertical lines with percentages indicate the percentage of descriptions with a coding time within that bracket.

The descriptive statistics of the coders’ timestamps can be found in table 1. The distribution of the timestamps for manual and OPERAS coding can be found in figure 1. Overall median coding time for manual coding was 60.6 s compared with 38.0 for OPERAS coding. For coder P1, the median coding times were 28.0 s for OPERAS and 62.0 s for manual coding. For P2, they were 48.2 and 81.3 s; for P3, 27.0 and 39.0 s; for P4, 42.9 and 63.5 s; and for P5, 43.2 and 66.5 s for OPERAS and manual coding, respectively. OPERAS coding demonstrated a faster overall median coding time of 22.6 s (t(5)=6.542, p=0.003). This is also the case for individual coders, with faster median coding times of 34 s for P1 (Z=4.072, p<0.001), 33.1 s for P2 (Z=2.298, p=0.022), 12 s for P3 (Z=2.017, p=0.044), 20.6 s for P4 (Z=4.597, p<0.001) and 23.3 s for P5 (Z=4.133, p<0.001).

**Table 1:** Descriptive statistics of the coding time of occupational coding per individual job description in seconds

Coder	Condition	Min	Max	Mean (SE)	IQR (25%)	IQR (75%)
Overall	OPERAS	0.0	649.6	62.9 (2.2)	16.0	80.0
	Manual	0.0	735.0	83.5 (2.5)	27.0	109.1
P1	OPERAS	0.0	598.0	57.7 (4.6)	13.2	73.8
	Manual	0.0	653.0	71.8 (4.7)	20.0	98.0
P2	OPERAS	0.4	650.0	82.8 (7.2)	19.8	112.9
	Manual	0.0	513.5	108.5 (7.6)	31.4	145.3
P3	OPERAS	1.0	226.8	31.7 (3.4)	11.0	52.0
	Manual	1.0	398.0	48.8 (4.7)	21.0	56.0
P4	OPERAS	1.0	596.5	68.5 (4.3)	18.0	92.5
	Manual	3.0	562.0	86.0 (4.6)	29.0	113.5
P5	OPERAS	0.0	363.0	59.9 (3.8)	14.0	80.3
	Manual	1.0	735.0	92.7 (6.5)	29.0	117.7

Following the within-subject design, each participant coded both using OPERAS and manually. P1–P5 refers to coders’ coding time. IQR is given for 25% and 75% quantiles. SE refers to the SE of the mean.

**Figure 2:**
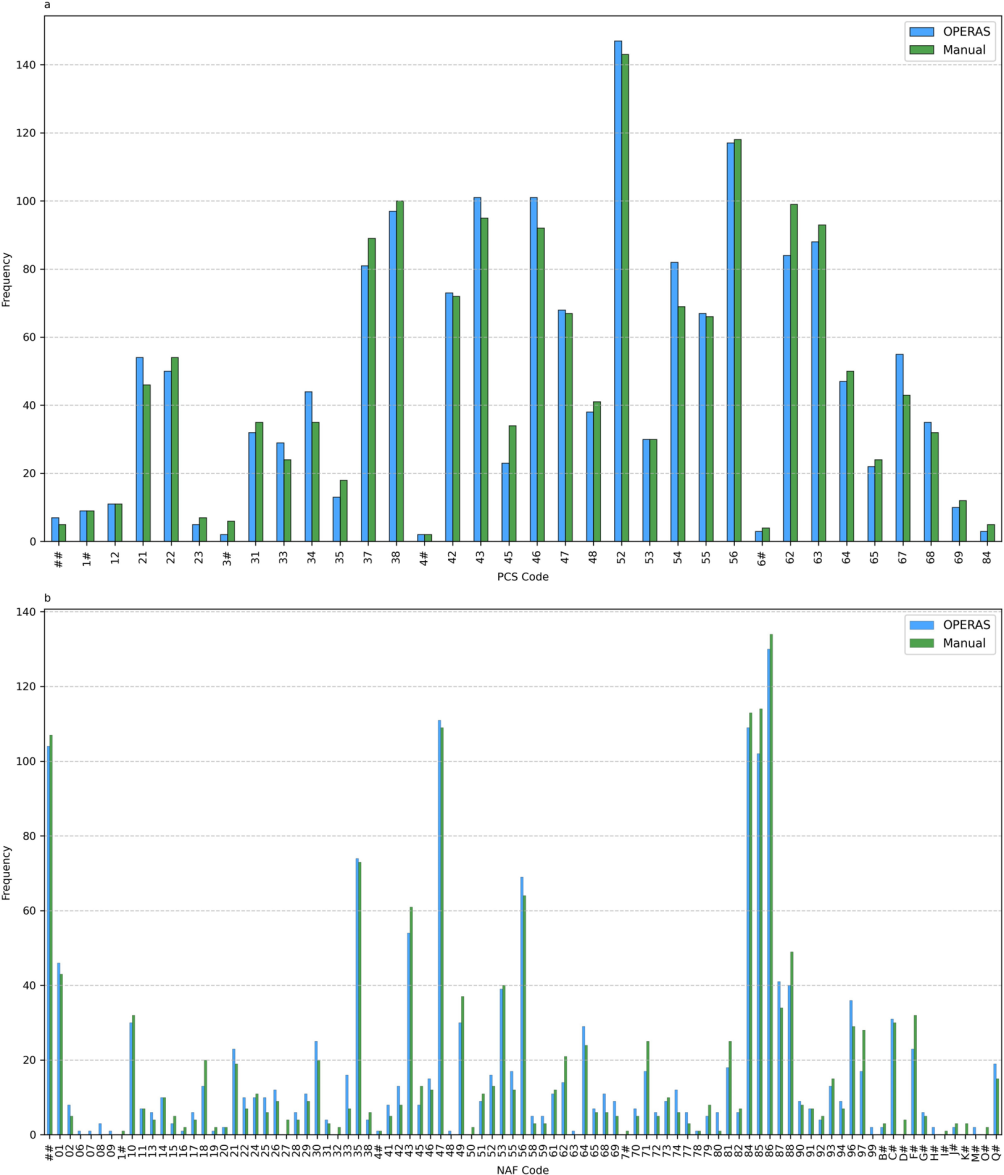
Distribution of second-level (a) PCS and (b) NAF codes selected by expert coders during OPERAS and manual coding. A ‘#’ in a PCS or NAF code indicates an uncodable coding level due to missing or incomplete job description information.

Inter-coder reliability for each possible coder pair on all coding levels can be found in table 2. For the PCS, the inter-coder reliability ranged 0.80–0.87, 0.73–0.83, 0.68–0.76, 0.61–0.70 for OPERAS coding, while for manual coding this ranged 0.80–0.85, 0.72–0.76, 0.66–0.72, 0.56–0.61 on coding levels 1–4, respectively. For the NAF, this ranged 0.70–0.85, 0.67–0.83, 0.45–0.70, 0.39–0.65 and 0.38–0.61 for OPERAS coding and 0.69–0.82, 0.66–0.77, 0.47–0.68, 0.39–0.62, 0.34–0.61 for manual coding on coding levels 1–5, respectively. Out of nine comparisons, OPERAS outperformed manual coding in eight instances on the first and second coding level, and in all instances for the third and fourth coding levels. For the NAF, OPERAS outperformed manual coding in seven instances for the first coding level, eight instances for the second through fourth coding levels, and in all instances for the fifth coding level. No significant differences were observed in the code distributions between OPERAS and manual coding for second-level PCS (χ²(33, N=3260)=14.34, p=0.99) and NAF codes (χ²(92, N=3260)=77.03, p=0.86) (see figure 2).

**Table 2:** For each coder pair (CP), the inter-coder reliability of assigning occupation (PCS) and activity sector (NAF) codes per coding level (CL) in Cohen’s kappa (k) for OPERAS coding and manual coding is given

CP	CL	PCS		NAF	
		OPERAS	Manual	OPERAS	Manual
P1 and P4	1	**0.85**	0.84	0.70	**0.72**
	2	**0.80**	0.76	**0.67**	0.66
	3	**0.74**	0.66	**0.50**	0.47
	4	**0.64**	0.56	**0.42**	0.39
	5	–	–	**0.39**	0.34
P2 and P3	1	**0.84**	0.81	**0.77**	0.73
	2	**0.81**	0.74	**0.77**	0.68
	3	**0.72**	0.67	**0.61**	0.61
	4	**0.67**	0.56	**0.55**	0.51
	5	–	–	**0.52**	0.50
P2 and P5	1	**0.87**	0.80	**0.83**	0.82
	2	**0.83**	0.75	**0.82**	0.76
	3	**0.76**	0.67	**0.70**	0.68
	4	**0.70**	0.59	**0.65**	0.62
	5	–	–	**0.61**	0.61
P3 and P5	1	**0.84**	0.81	**0.85**	0.79
	2	**0.81**	0.76	**0.83**	0.77
	3	**0.75**	0.72	**0.67**	0.62
	4	**0.68**	0.61	**0.63**	0.58
	5	–	–	**0.59**	0.55
P1 and GS	1	**0.85**	0.85	0.70	**0.77**
	2	**0.77**	0.75	0.67	**0.71**
	3	**0.73**	0.66	0.45	**0.48**
	4	**0.65**	0.59	0.39	**0.42**
	5	–	–	**0.38**	0.36
P2 and GS	1	**0.83**	0.81	**0.81**	0.72
	2	**0.74**	0.73	**0.79**	0.67
	3	**0.68**	0.66	**0.63**	0.56
	4	**0.61**	0.60	**0.60**	0.47
	5	–	–	**0.56**	0.46
P3 and GS	1	0.80	**0.81**	**0.77**	0.69
	2	0.73	**0.74**	**0.75**	0.67
	3	**0.69**	0.68	**0.60**	0.52
	4	**0.62**	0.57	**0.56**	0.46
	5	–	–	**0.50**	0.44
P4 and GS	1	**0.86**	0.82	0.72	**0.77**
	2	**0.79**	0.73	0.70	**0.72**
	3	**0.73**	0.67	**0.60**	0.58
	4	**0.64**	0.57	**0.55**	0.54
	5	–	–	**0.54**	0.50
P5 and GS	1	**0.86**	0.81	**0.78**	0.74
	2	**0.78**	0.72	**0.78**	0.71
	3	**0.72**	0.66	**0.65**	0.58
	4	**0.65**	0.60	**0.59**	0.54
	5	–	–	**0.56**	0.53

Following the within-subject design, each participant coded both using OPERAS and manually. GS refers to the gold standard manually coded job episode from CONSTANCES. A number is bold if the k-value exceeds the other condition for that coder pair and classification.

The average score for individual SUS questions ranged between 1.2 and 4.8, with the SUS score from coders ranging 65–87.5 (see table 3). The average SUS score is 75.5.

**Table 3:** System Usability Scale (SUS) questions on OPERAS’ perceived usability and scores for coders 1–5 (P1–P5) and average scores (Avg)

No.	Question	P1	P2	P3	P4	P5	Avg
1	I think that I would like to use this system frequently	5	3	5	2	3	3.6
2	I found the system unnecessarily complex	1	2	1	1	1	1.2
3	I thought the system was easy to use	5	5	5	5	4	4.8
4	I think that I would need the support of a technical person to be able to use this system	1	1	1	1	2	1.2
5	I found the various functions in this system were well integrated	4	2	3	3	4	3.2
6	I thought there was too much inconsistency in this system	3	3	1	4	5	3.2
7	I would imagine that most people would learn to use this system very quickly	4	4	5	3	4	4
8	I found the system very cumbersome to use	1	2	1	3	2	1.8
9	I felt very confident using the system	4	3	4	3	2	3.2
10	I needed to learn a lot of things before I could get going with this system	1	2	1	1	1	1.2
	**SUS score**	87.5	67.5	92.5	65	65	75.5

Coder scores are on a Likert scale from 1 (strongly disagree) to 5 (strongly agree). SUS scores are calculated by subtracting one from odd-numbered question scores, subtracting even-numbered question scores from 5, summing the results and subsequently multiplying by 2.5.

## Discussion

In this study, we quantitatively assess the impact of the DSS OPERAS on occupational coding time and inter-coder reliability of assigning occupation and activity sector codes to job descriptions. Five expert coders proficient in coding using the PCS2003 and NAF2008 participated in the study. Using a within-subject design, occupational coding performance using OPERAS was compared with traditional manual coding. OPERAS was shown to benefit both coding time and inter-coder reliability.

OPERAS consistently outperformed manual coding, both overall and for each coder individually. Differences in coder performance (see table 1) can be attributed to differences in prior coding experience, familiarity with the classification systems and utilisation of OPERAS’ features. For example, the hover function, which displays a code suggestion’s corresponding coding index information, was not fully used by all participants. This led to manual checks of each suggested code’s coding index entry, resulting in substantial time loss. Furthermore, none of the coders used the automatic coding function, despite its significant potential to enhance coding time.^13^ OPERAS’ original data processing and classification models are used, achieving prediction accuracies of 68.8% and 78.9%, resulting in potential workload reductions of 40.7%–55.7% for PCS2003 and NAF2008, respectively.^13^ Despite its state-of-the-art performance compared with other tools, which have prediction accuracies ranging 15%–65%,^69^,^11 13 20 21 30^ users were still hesitant to employ the automatic coding function. This reluctance likely stems from the coders’ initial lack of trust in OPERAS’ recommendations due to first-time usage.^31^ Consequently, all coders verified each suggestion’s accuracy before accepting, regardless of confidence score, resulting in higher coding time. Overall, with continued OPERAS usage and increased trust in the system, coders are expected to become more likely to fully use OPERAS’ functionalities and accept high-confidence suggestions, leading to progressively improving coding time.^31^

On the least aggregated coding level, OPERAS outperforms manual coding. This outcome was expected because users are more likely to select a suggested code than to independently agree on the same code without suggestions.^21 32^ Increased inter-coder reliability leads to more stable and accurate results.^33^ Therefore, when suggestions are validated, receiving suggestions from DSS is preferred for occupational epidemiological studies and other research based on these occupational codes. No significant differences were observed in the distribution of second-level PCS and NAF codes between OPERAS and manual coding. This finding suggests that the recommendations provided by OPERAS do not lead to code choices that significantly deviate from those manually selected, thereby demonstrating comparable reliability. This is further supported by the percentage of corrections made by participants during OPERAS coding: P1 corrected 33.7% of PCS codes and 47.9% of NAF codes, P2 corrected 36.2% and 45.4%, P3 corrected 33.7% and 44.5%, P4 corrected 33.7% and 44.5%, and P5 corrected 43.9% and 57.1%, respectively. These results highlight that, although some incorrect codes were initially presented to the expert coders, final choices were not influenced by these errors and often aligned with the codes that would have been selected during manual coding.

OPERAS did not outperform manual coding on inter-coder reliability in all cases. In 9.8% of cases (ie, 8 out of 81), manual coding surpassed OPERAS coding. This could be attributed to various factors, such as differences and similarities between coder pairs in terms of training and experience.^34 35^ Furthermore, in the current study, no specific end goal was defined for which the resulting codes will be used. In cases where certain exposed job groups require thorough and/or additional review due to high exposure risk, OPERAS’ models could be adapted to ensure higher reliability for these codes.

In all instances, expert inter-coder reliability using the NAF was lower than when using PCS. This was expected given that NAF has more outcome categories.^36 37^ Additionally, job descriptions were selected if the gold-standard PCS code in CONSTANCES was coded to at least the second coding level. Consequently, the gold-standard NAF was sometimes coded to even lower levels due to insufficient job description information, potentially leading to increased variability and ambiguity compared with PCS coding.^38^

With an average SUS score of 75.5, OPERAS’ perceived usability is considered ‘good’.^24^ However, it is notable that P4 and P5, who reported the lowest usability scores (ie, 65), experienced the greatest coding time gains. This indicates potential for further improvement, as OPERAS usage improved coding time even when perceived usability was low. For example, OPERAS was sometimes considered inconsistent. Integrating explainability into OPERAS could address the perceived inconsistency by providing rationale for code suggestions, potentially reducing user uncertainty and improving trust.^39^ Beyond clarifying suggestions, this explainability could also enable respondent self-coding, which already demonstrates reasonable reliability and efficiency when respondents traverse a decision tree to select their occupation.^40^ Here, OPERAS’ suggestions and explanations can be leveraged to guide users in this selection process, selecting the occupational category and coding level they deem most appropriate. Furthermore, the underutilisation of OPERAS’ hover function suggests that this, and possibly other features, may not yet be effectively implemented or integrated (see table 3). Therefore, usability studies and other qualitative evaluations (eg, interviews) should be conducted to identify and address bottlenecks in OPERAS’ usability to increase usability and potentially maximise coding time gains.

The primary cause of data loss was due to the absence or corruption of parts of the video files for both conditions, which led to a total data loss of 26.2%. Despite this, the impact on the overall results was minimised because no specific parts of the subsets were consistently affected. Additionally, manual corrections of timestamps ensured that only valid timestamps were analysed, securing accurate and stable results. Furthermore, a comparison conducted with complete, uncleaned data yielded similar inferences.

This study involved five expert coders proficient with the PCS2003 and NAF2008 coding systems. As different classification systems can influence coding workflows, which may also vary among expert coders, future comparison studies could include a larger number of coders who are familiar with these and other occupational coding classifications. This study demonstrates that using a DSS for occupational coding decreases coding time and improves inter-coder reliability compared with traditional manual coding. Additional insights could be gained by conducting a similar study with more coders and diverse classification systems. Furthermore, OPERAS’ coding time and inter-coder reliability gain could be further improved by conducting usability studies to find and solve potential bottlenecks in OPERAS’ usability. Overall, OPERAS may facilitate large-scale, harmonised job coding, potentially enabling more stable and efficient occupational health research.

## Supplementary material

10.1136/oemed-2024-109823 online supplemental file 1

## Data Availability

Data are available upon reasonable request.
