# Supplementary material for: OPERAS decision support system versus manual job coding: a quantitative analysis on coding time and inter-coder reliability
Source: Occup Environ Med. 2025 Jun 13;82(4):e109823. doi: 10.1136/oemed-2024-109823 (PMC12322435; doi:10.1136/oemed-2024-109823)
Supplement: online supplemental file 1 [file oemed-82-4-s001.docx]

**Supplemental Material**


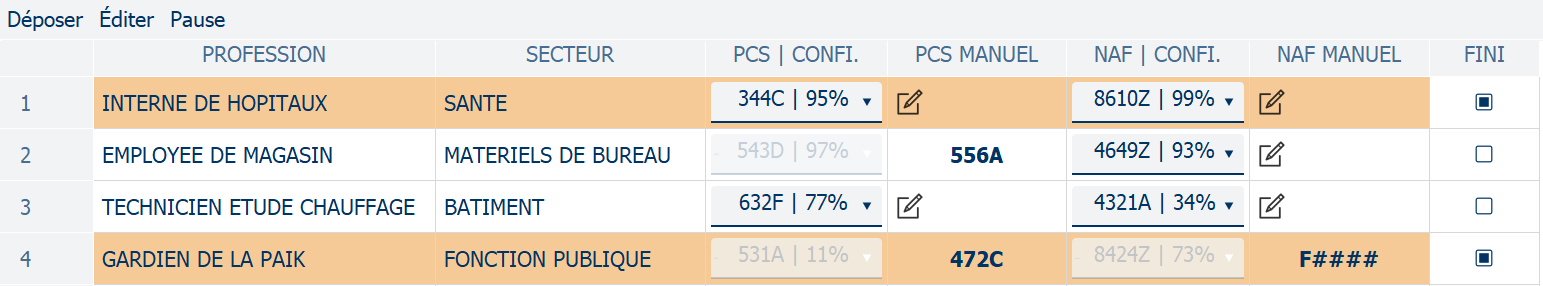


Supplementary Figure S1: Screenshot of the first 4 rows of OPERAS’ user interface. Each row shows a job description, dropdown menu with OPERAS’ suggested codes, manual coding field for expert correction, and checkbox to indicate a job description has been coded.
